# Limited proteolysis of human histone deacetylase 1

**DOI:** 10.1186/1471-2091-7-22

**Published:** 2006-10-05

**Authors:** Nayana Kamath, Paulina Karwowska-Desaulniers, Mary Kay H Pflum

**Affiliations:** 1 Department of Chemistry, Wayne State University, Detroit, MI 48202, USA

## Abstract

**Background:**

Histone deacetylase (HDAC) proteins are associated with cell proliferation, differentiation, apoptosis, and cancer. Specifically, HDAC1 is linked with cell growth, a hallmark of cancer formation. HDAC1 is a phosphoprotein and phosphorylation at S421 and S423 promotes HDAC1 enzymatic activity and protein association. While single and double point mutants of HDAC1 at S421 and S423 appear functionally similar, the evidence suggests that HDAC1 is phosphorylated simultaneously at both S421 and S423 *in vivo*. Additional experiments are necessary to probe the role of double phosphorylation of HDAC1 at S421 and S423.

**Results:**

To characterize HDAC1 phosphorylation at S421 and S423, limited proteolysis of HDAC1 was performed for the first time. HDAC1 degraded without production of discrete fragments. By performing concentration-dependent proteolysis, HDAC1 double point mutants with disrupted phosphorylation at S421 and S423 displayed different trypsin sensitivities compared to wild type HDAC1. Unexpectedly, HDAC1 single point mutants with disrupted phosphorylation at either S421 or S423 demonstrated protease sensitivity similar to the wild type HDAC1.

**Conclusion:**

Concentration-dependent proteolysis experiments provide evidence that phosphorylation of S421 and S423 individually contribute to HDAC1 function. In addition, the limited proteolysis experiments support a model where associated proteins promote HDAC1 enzymatic activity, reinforcing the importance of protein interactions in HDAC1 structure and function. Finally, because HDAC1 does not display distinct regions of protease sensitivity, the proteolysis studies suggest that HDAC1 comprises inter-related structural regions.

## Background

Histone deacetylase (HDAC) proteins play a critical role in regulating gene expression *in vivo *by altering the accessibility of genomic DNA to transcription factors. Specifically, HDAC proteins remove the acetyl group of acetyl-lysine residues on histones, which can result in nucleosomal remodelling [1]. Due to their governing role in gene expression, HDAC proteins are associated with a variety of cellular events, including cell cycle regulation, cell proliferation, differentiation and cancer development [2-5]. In fact, HDAC inhibitors reduce tumour growth in various human tissues, including lung, stomach, breast, and prostrate [6]. As a result, small molecule inhibitors of HDAC enzymatic activity are currently being exploited as potential cancer drugs [7-9].

The study of HDAC proteins in cancer is complicated by the identification of eleven human HDAC proteins that are sensitive to small molecule inhibitors [10]. Although it is unclear which of the eleven HDAC proteins is involved in cancer formation, the activity of HDAC1 has been linked to cell proliferation, a hallmark of cancer. Particularly, mammalian cells with knock down of HDAC1 expression using siRNA were antiproliferative [11]. While the knock out mouse of HDAC1 was embryonic lethal, the resulting stem cells displayed altered cell growth [12]. Mouse cells overexpressing HDAC1 demonstrated a lengthening of G_2 _and M phases and reduced growth rate [13]. Therefore, the reported data implicate HDAC1 in cell cycle regulation and cell proliferation.

To further elucidate HDAC1 function *in vivo*, HDAC1 associated proteins have been characterized by biochemical purification. HDAC1 exists in at least three distinct biochemical complexes. The NuRD complex includes a core complex comprised of HDAC1, HDAC2, retinoblastoma associated protein 46 (RbAp46), RbAp48, as well as Mi2, methyl CpG binding domain 3 (MBD3) and metastasis associated protein 2 (MTA2) [14-16]. The fact that the NuRD complex contains MTA2, which is associated with cancer metastasis, provides further evidence that HDAC1 may play a role in cancer development. The Sin3 complex comprises the core complex as well as mSin3, Sin3 associated protein 18 (SAP18), SAP30, and SDS3 [17-21]. Finally, the CoREST complex is less well characterized and contains HDAC1, HDAC2, CoREST, and p110 (KIAA0601) [22,23]. The presence of associated proteins promotes the enzymatic activity of HDAC1. Specifically, coexpression of MTA2 of the NuRD complex with the core HDAC1-containing complex resulted in augmented deacetylase activity [16]. Most recently, knockdown of SDS3 of the Sin3 complex resulted in reduced deacetylase activity of HDAC1 immunoprecipitates in mammalian cells [20].

HDAC1 protein association and activity are also promoted by phosphorylation. HDAC1 is phosphorylated at S421 and S423 [24], although additional phosphorylated sites are also possible [25,26]. Single or double mutation of S421 and S423 disrupted HDAC1 interactions with members of the Sin3, NuRD, and CoREST complexes, including RbAp46, mSin3A, MTA2, and CoREST [24]. In addition, single or double mutation of S421 and S423 reduced deacetylase activity. While single or double mutants of HDAC1 appear functionally similar, the evidence suggests that HDAC1 is phosphorylated at both S421 and S423 *in vivo *[24,25]. Additional experiments are necessary to reveal the functional significance of double phosphorylation of HDAC1.

To explore the role of phosphorylation in promoting HDAC1 activity and protein association, we have performed a limited trypsin proteolysis of HDAC1. Because the conformation and/or protein associations of HDAC1 are likely related to proteolysis sensitivity, we hypothesized that concentration-dependent trypsin digestion will change as a function of phosphorylation state. We found that HDAC1 mutants lacking phosphorylation at both S421 and S423 displayed different proteolysis sensitivities than wild type HDAC1. Interestingly, HDAC1 mutants lacking only one phosphorylated residue displayed proteolysis sensitivities similar to wild type HDAC1. The limited proteolysis study suggests that phosphorylation of S421 and S423 individually contribute to HDAC1 function.

## Results

### Limited trypsin digestion of endogenous HDAC1

**Figure 1:**
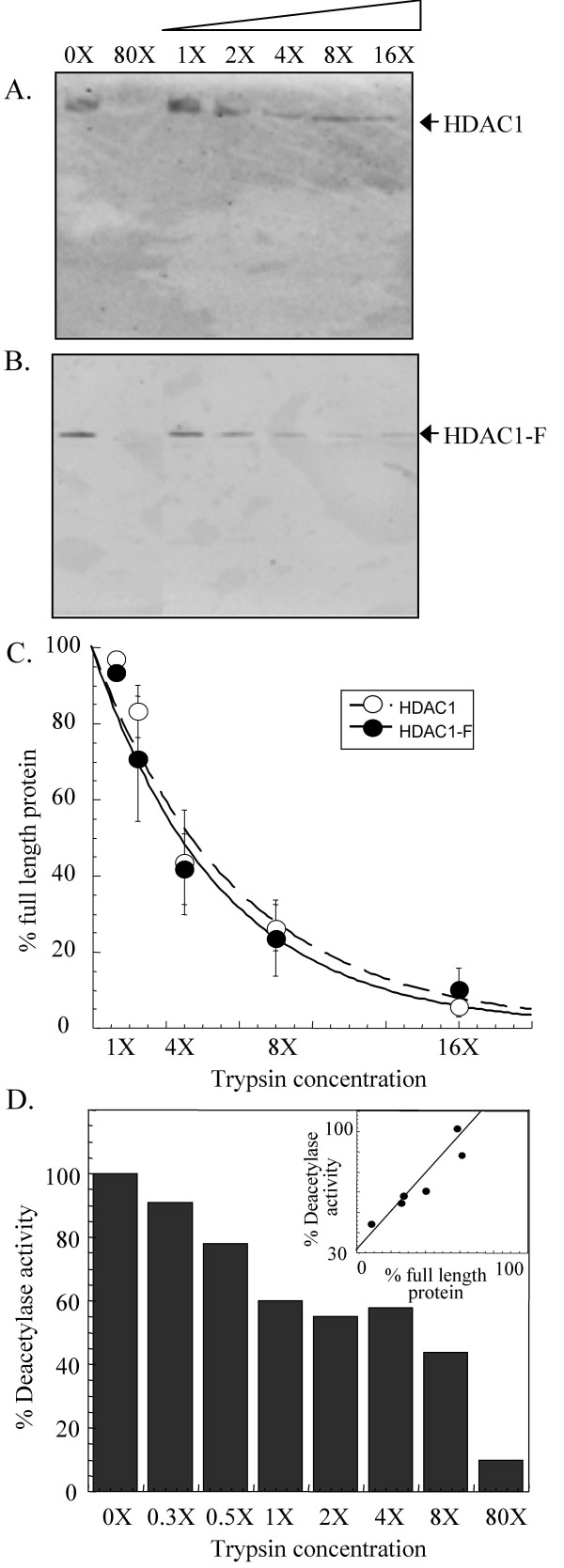
**Limited proteolysis of endogenous and transiently expressed HDAC1**. Immunoprecipitated endogenous HDAC1 (A) and transiently expressed HDAC1-Flag (HDAC1-F) (B) were incubated at room temperature with increasing concentrations of trypsin (0.000625 μg/μL- 1X, 0.00125 μg/μL- 2X, 0.0025 μg/μL- 4X, 0.005 μg/μL- 8X, 0.01 μg/μL- 16X, and 0.05 μg/μL- 80X). After separation by SDS-PAGE, the proteins were visualized with either anti-HDAC1 antibody in case of endogenous HDAC1 or anti-Flag antibody in case of transiently expressed HDAC1-F. (C) A graph showing percentage of full length endogenous HDAC1 (white) and transiently expressed HDAC1-Flag (black) remaining after exposure to different concentrations of trypsin. The curves were generated by least squares fit to a single exponential. (D) A plot displaying the deacetylase activity of HDAC1-F after incubation with an increasing concentration of trypsin. The inset displays the relationship between deacetylase activity and percentage of full length HDAC1-F remaining after exposure to increasing concentrations of trypsin and the data was fit to a linear equation.

As a first step towards characterizing the protease sensitivity of HDAC1, endogenous HDAC1 from human Jurkat cells was immunoprecipitated and incubated with increasing concentrations of trypsin (0.000625-0.01 μg/μL). The trypsin digestion products were separated using SDS-PAGE and HDAC1 was visualized using an anti-HDAC1 antibody recognizing the C-terminus (Figure 1A). For clarity, the lowest concentration of trypsin (0.000625 μg/μL) is indicated as 1X and, accordingly, the highest concentration (0.01 μg/μL) as 16X. A trypsin concentration of 80X (0.5 μg/μL) was also used to digest HDAC1 completely, as a positive control. Endogenous HDAC1 showed concentration-dependent cleavage by trypsin (Figure 1A). The concentration of trypsin required to degrade roughly half of the full-length protein was 4X (0.0025 μg/μL). Interestingly, western blot analysis did not show a protease fragmentation pattern with endogenous HDAC1; only full length HDAC1 was observed with all concentrations of trypsin. Because the HDAC1 antibody only recognizes the C-terminus of the protein, we also analyzed the reactions products by silver staining. Again, no protein fragments associated with HDAC1 degradation were observed (data not shown). Additional conditions including varying temperatures (4°C and 25°C) and varying reaction times (10 seconds to 30 minutes) were tested with only full length HDAC1 observed (data not shown). The results suggest that HDAC1 is digested by trypsin to short peptide fragments, which are not identifiable by gel methods.

**Figure 2:**
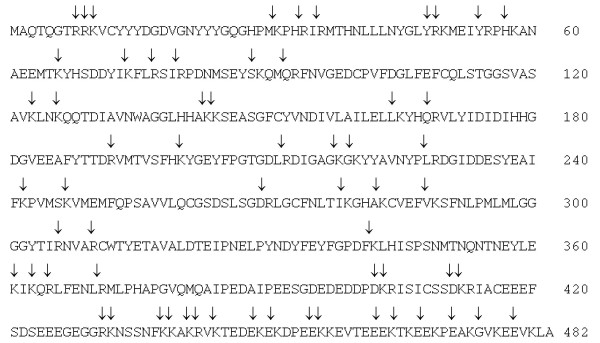
**Predicted trypsin cleavage sites of HDAC1**. The primary sequence of HDAC1 (accession number U50079) is shown with arrows indicating the trypsin cleavage sites predicted using the Peptide Cutter program [27]. More information on the sequences, lengths, masses, and cleavage probabilities of the predicted peptide fragments are available [see Additional File 1].

To support the observation that HDAC1 degrades to short peptide fragments in the presence of trypsin, the predicted sites of HDAC1 trypsin cleavage were determined using the program Peptide Cutter [27]. As shown in Figure 2, 62 trypsin cleavage sites were identified with the largest likely fragments to be 32 amino acids with a mass of roughly 3.8 kDa [see Additional File 1]. The predicted tryptic map of HDAC1 corroborates the proteolysis results suggesting that HDAC1 degrades to small peptide fragments in the presence of trypsin.

### Limited trypsin digestion of transiently expressed HDAC1

In preparation for testing HDAC1 phosphorylation site mutants, we first validated the use of transiently expressed HDAC1-Flag fusion protein (HDAC1-F) in concentration-dependent proteolysis experiments. Immunoprecipitated HDAC1-F was treated with increasing concentrations of trypsin, as described for endogenous HDAC1. Like endogenous HDAC1, roughly half of the protein was degraded with 4X concentration of trypsin (Figure 1B). Therefore, the data revealed that HDAC1-F demonstrated similar concentration-dependent cleavage by trypsin as endogenous HDAC1.

To allow a more rigorous comparison of the concentration-dependent cleavage, the amount of full-length protein present after incubation with each concentration of trypsin was quantified and compared to the amount of protein present in the absence of trypsin (Figure 1C). Consistent with visual observations, 44 ± 14% of endogenous HDAC1 and 42 ± 9.3% of expressed HDAC1-F remained in the presence of 4X trypsin [see Additional File 2]. The quantitative analysis reinforces the conclusion that endogenous HDAC1 and expressed HDAC1-F display similar concentration-dependent degradation by trypsin.

Like with endogenous HDAC1, no proteolytic fragments of HDAC1-F were observed in these studies (Figure 1B), consistent with the production of small peptides upon degradation. To corroborate the possibility that complete HDAC1 degradation occurs with proteolysis, we assessed the enzymatic activity of HDAC1 after incubation with increasing concentrations of trypsin (Figure 1D). Like the proteolysis experiments, HDAC1 enzymatic activity was reduced in a trypsin concentration-dependent manner. The linear relationship between trypsin proteolysis and enzymatic activity (see inset in Figure 1D) is consistent with the scenario that HDAC1 degrades to small peptide fragments in the presence of trypsin.

### Limited trypsin digests of HDAC1 phosphorylation site double mutants

To probe the influence of HDAC1 phosphorylation on trypsin sensitivities, two HDAC1 mutants with disrupted phosphorylation at S421 and S423 were tested- the HDAC1 S421A/S423A double mutant lacks the serine hydroxyl groups that are phosphorylated and the HDAC1 E424A/E426A double mutant lacks the CK2 recognition sequence required for phosphorylation *in vivo*. Previous work showed that the HDAC1 S421A/S423A and HDAC1 E424A/E426A mutants behave similarly; both mutants are unable to interact with RbAp48, Sin3A, MTA2, and CoREST and are catalytically inactive, demonstrating 22.9 ± 3.1 % and 30.9 ± 1.6 % of wild type activity, respectively [24].

**Figure 3:**
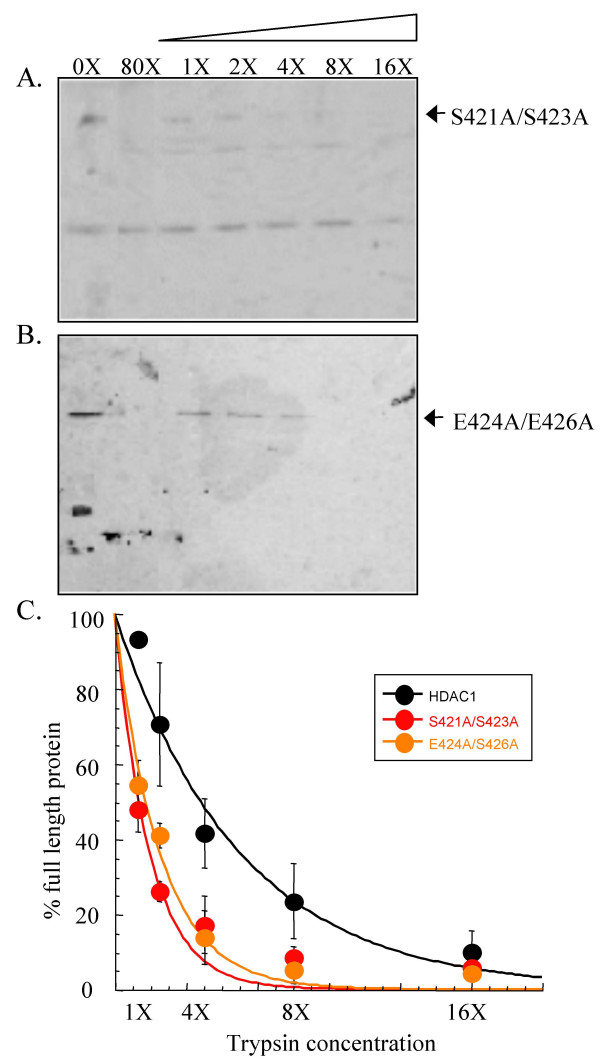
**Limited proteolysis of HDAC1 S421A/S423A and HDAC1 E424A/E426A mutants**. Immunoprecipitated HDAC1 S421A/S423A (A) and HDAC1 E424A/E426A (B) mutants were incubated with increasing concentrations of trypsin (see Figure 1), separated by SDS-PAGE, and visualized with anti-Flag antibody. The faster migrating proteins present in the absence of trypsin (0X) are derived from the anti-Flag-bound solid phase used for immunoprecipitation [see Additional File 3]. (C) A graph showing percentage of full length S421A/S423A mutant (red) and E424A/E426A mutant (orange) remaining after exposure to different concentrations of trypsin. The data with wild type HDAC1-F (black- Figure 1) is also included for comparison. The curves were generated by least squares fit to a single exponential.

Transiently expressed HDAC1 S421A/S423A and HDAC1 E424A/E426A mutants were incubated with increasing concentrations of trypsin, as described for the HDAC1-F (Figure 3A and 3B). Quantification of the western blot analysis (Figure 3C) revealed that HDAC1 S421A/S423A and HDAC1 E424A/E426A mutants display 17 ± 7.6 % and 14 ± 7.2 % of full-length protein, respectively, in the presence of 4X trypsin [see Additional File 2]. The experiments demonstrated that the HDAC1 S421A/S423A and HDAC1 E424A/E426A mutant proteins display equivalent concentration-dependent trypsin digestion, consistent with previous results showing that they maintain similar deacetylase activity and complex forming ability [24].

While the HDAC1 S421A/S423A and HDAC1 E424A/E426A mutants maintain similar trypsin sensitivities, they demonstrated significant differences in concentration-dependent trypsin digestion compared to wild-type HDAC1 (Figure 3C). Quantification revealed that 42 ± 9.3 % of wild-type protein compared with 17 ± 7.6 % of HDAC1 S421A/S423A and 14 ± 7.2 % of HDAC1 E424A/E426A mutants was observed in the presence of 4X trypsin [see Additional File 2]. Even more dramatically, the HDAC1 S421A/S423A and HDAC1 E424A/E426A mutant proteins were sensitive to degradation with 1X trypsin (48 ± 6.0 % and 55 ± 6.4 %, respectively), whereas the wild-type HDAC1 was almost completely insensitive to degradation at that concentration (93 ± 1.3 %). The data show that the HDAC1 S421A/S423A and HDAC1 E424A/E426A mutants are more sensitive to trypsin digestion than wild-type HDAC1.

### Limited trypsin digests of HDAC1 phosphorylation site single point mutants

Similar to the HDAC1 S421A/S423A double mutant, single point HDAC1 S421A and HDAC1 S423A mutants are catalytically inactive, demonstrating 33 ± 4.2 % and 25 ± 2.9 % activity compared to wild type HDAC1, respectively [24]. In addition, HDAC1 S421A/S423A, S421A and S423A mutants similarly lack the ability to bind RbAp48, mSin3, MTA2, and CoREST. Because the single and double point mutants display similar activities and protein associations, the expectation was that the S421A and S423A single point mutants would demonstrate similar trypsin sensitivities compared to the HDAC1 S421A/S423A double mutant.

**Figure 4:**
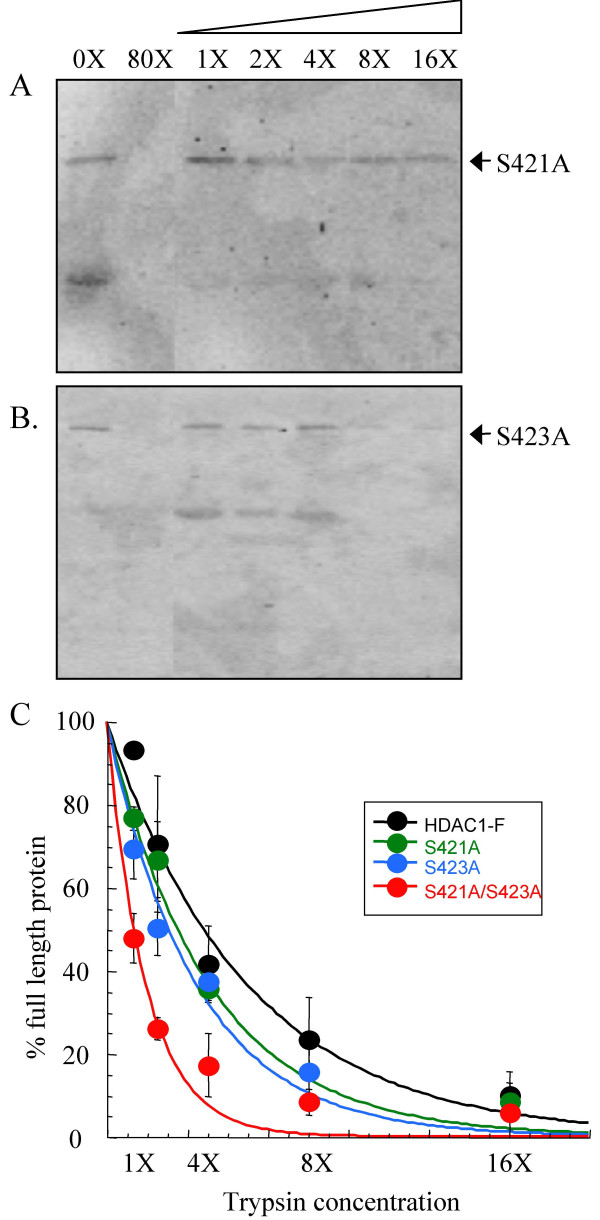
**Limited proteolysis of HDAC1 S421A and HDAC1 S423A mutants**. Immunoprecipitated HDAC1 S421A (A) and HDAC1 S423A (B) mutants were incubated with increasing concentrations of trypsin (see Figure 1), separated by SDS-PAGE, and visualized with anti-Flag antibody. The faster migrating proteins present in the absence of trypsin (0X) are derived from the anti-Flag-bound solid phase used for immunoprecipitation [see Additional File 3]. (C) A graph showing percentage of full-length HDAC1 S421A (green) and HDAC1 S423A (blue) mutants remaining after exposure to different concentrations of trypsin. The data with wild type HDAC1-F (black- Figure 1) and HDAC1 S421A/S423A (red- Figure 2) proteins are also included for comparison. The curves were generated by least squares fit to a single exponential.

Concentration-dependent trypsin digestion was performed with transiently expressed HDAC1 S421A and HDAC1 S423A, as described previously (Figure 4A and 4B). Quantitative analysis of the western blots (Figure 4C) indicated that both mutants displayed similar amounts of full-length protein at every concentration of trypsin [see Additional File 2]. Whereas HDAC1 S421A and HDAC1 S423A displayed nearly identical concentration-dependent trypsin digestion sensitivities, they demonstrated significantly different trypsin sensitivities compared to the HDAC1 S421A/S423A double mutant. While the HDAC1 S421A/S423A double mutant was almost completely degraded at 4X trypsin concentration (17 ± 7.6 %), the HDAC1 S421A and S423A mutants were only partially degraded (36 ± 2.9 % and 38 ± 3.7 %, respectively). Instead, the S421A and S423A single point mutants displayed similar trypsin sensitivities to the wild type HDAC1-F protein. For example, while HDAC1-F was partially degraded (42 ± 9.3 %) with 4X trypsin concentration, the HDAC1 S421A and HDAC1 S423A single point mutants were also partially degraded (36 ± 2.9 % and 38 ± 3.7 %, respectively). In fact, only with 1X trypsin concentration did HDAC1 S421A and HDAC1 S423A single point mutants show differences compared with wild-type HDAC1; the single point mutants displayed 77 ± 2.8% or 70 ± 7.0% full length protein, which is roughly of an intermediate degradation compared to wild-type HDAC1 (93 ± 1.3 %) or HDAC1 S421A/S423A (48 ± 6 %). Therefore, the comparison revealed an unexpected similarity between the trypsin sensitivities of wild type HDAC1-F and the HDAC1 S421A and HDAC1 S423A single point mutants.

### Limited trypsin digests of HDAC1 kinase consensus site single point mutants

To reinforce the observations with HDAC1 S421A and HDAC1 S423A single point mutants, the CK2 consensus site single point mutant HDAC1 E424A and HDAC1 E426A were also studied. Like HDAC1 S421A and HDAC1 S423A, mutation of E424 and E426 resulted in reduced enzymatic activity, demonstrating 79 ± 5.4 % and 48 ± 7.2 % of wild type activity, respectively [24]. Unlike HDAC1 S421A and HDAC1 S423A, the HDAC1 E424A and HDAC1 E426A mutants bind to RbAp48 and mSin3, although only E424A binds with comparable affinity to the wild-type HDAC1 protein. Because HDAC1 S421A and HDAC1 S423A mutants demonstrated similar trypsin sensitivities to wild type HDAC1, the expectation was that HDAC1 E424A and HDAC1 E426E would also.

**Figure 5:**
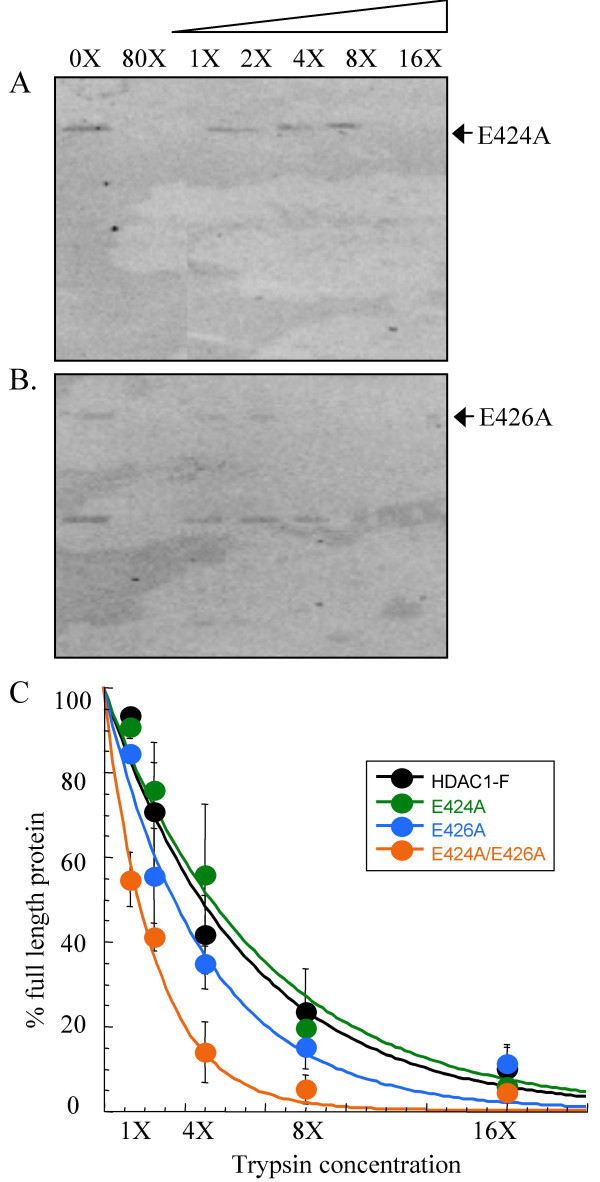
**Limited proteolysis of HDAC1 E424A and HDAC1 E426A mutants**. Immunoprecipitated HDAC1 E424A (A) and HDAC1 E426A (B) mutants were incubated with increasing concentrations of trypsin (see Figure 1), separated by SDS-PAGE, and visualized with anti-Flag antibody. The faster migrating proteins present in the absence of trypsin (0X) are derived from the anti-Flag-bound solid phase used for immunoprecipitation [see Additional File 3]. (C) A graph showing percentage of full length HDAC1 E424A (green) and HDAC1 E426A (blue) mutants remaining after exposure to different concentrations of trypsin. The data with wild type HDAC1-F (black- Figure 1) and HDAC1 E424A/E426A (orange- Figure 2) proteins are also included for comparison. The curves were generated by least squares fit to a single exponential.

Concentration-dependent trypsin digestion was performed with the HDAC1 E424A and HDAC1 E426A single point mutant (Figure 5A and 5B) and western blot analysis indicated that both mutants degraded similarly to HDAC1-F (Figure 5C) [see Additional Files 2]. While HDAC1-F was partially degraded (42 ± 9.3 %) with 4X trypsin concentration, the HDAC1 E424A and HDAC1 E426A single point mutants were also partially degraded (56 ± 17% and 35 ± 6.1%, respectively). While E424A was degraded to the same extent as wild-type HDAC1 at 1X trypsin (93 ± 1.3 % versus 91 ± 2.5 %, respectively), E426A displayed slightly reduced digestion compared to wild-type HDAC1 (84 ± 1.2 %). The data with HDAC1 E424A and HDAC1 E426A are consistent with studies of HDAC1 S4231A and HDAC1 S423A, demonstrating similar trypsin sensitivities compared with wild type HDAC1.

### Limited trypsin digestion of H141A HDAC1 mutant

**Figure 6:**
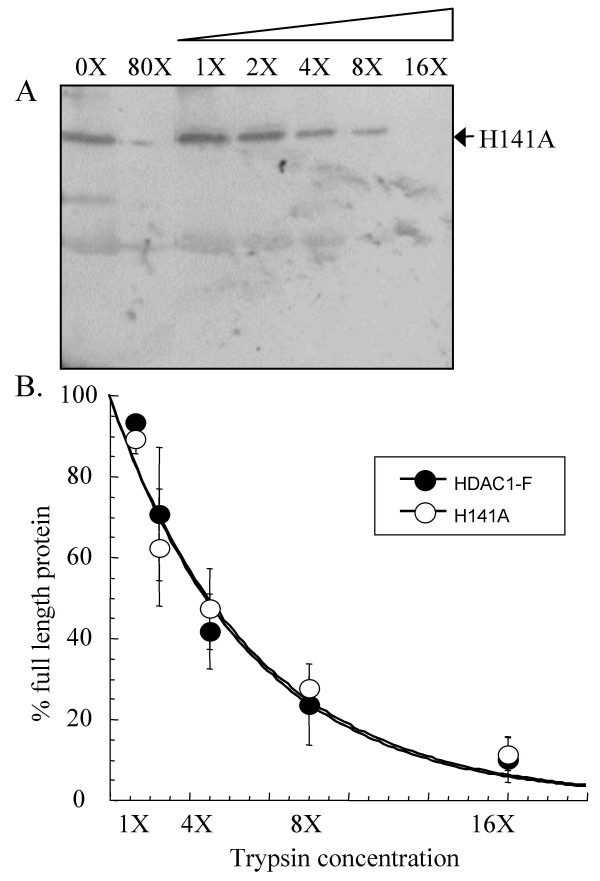
**Limited proteolysis of HDAC1 H141A mutant**. Immunoprecipitated HDAC1 H141A mutant (A) was incubated with increasing concentrations of trypsin (see Figure 1), separated by SDS-PAGE, and visualized with anti-Flag antibody. The faster migrating proteins present in the absence of trypsin (0X) are derived from the anti-Flag-bound solid phase used for immunoprecipitation [see Additional File 3]. (B) A graph showing percentage of full-length HDAC1 H141A mutant (white) remaining after exposure to different concentrations of trypsin. The data with wild type HDAC1-F (black- Figure 1) are also included for comparison. The curves were generated by least squares fit to a single exponential.

In addition to probing the phosphorylation-dependent changes in HDAC1 conformations, we explored the use of limited proteolysis to assess the conformational changes of a HDAC1 catalytic site mutant. Crystallography of human HDAC8 and two HDAC homologs from bacteria (HDLP and HDAH) have implicated a metal ion along with two conserved histidines in HDAC catalytic activity [28-31]. Consistent with the proposal, mutation of H141 results in loss of HDAC1 deacetylase activity [32]. Although the H141A mutant is catalytically inactive, it maintains full binding to RbAp48 and mSin3. To determine if trypsin sensitivities are altered by catalytic amino acid mutation, we tested the concentration-dependent trypsin digestion of the HDAC1 H141A mutant (Figure 6A). The trypsin degradation of HDAC1 H141A was identical to that of HDAC1-F at every concentration of trypsin tested (Figure 6B) [see Additional Files 2]. The data indicate that trypsin sensitivities are not altered by catalytic amino acid mutation, which influence enzymatic activity but not protein associations.

## Discussion

Structural insight into human HDAC1 is lacking. NMR, CD, or crystallographic characterization have been challenging due to the low quantities and contamination with associated proteins typical of mammalian cell-derived HDAC1 [33]. In addition, most HDAC proteins, including HDAC1, cannot be isolated in active form from bacteria [33]. Unlike other methods, limited proteolysis assays allow analysis of HDAC1 from mammalian sources because low concentrations and the presence of associated proteins are tolerated [34]. In addition, HDAC1 isolated from mammalian cells can be probed in its natural state *in vivo*, including its various states of post-translational modification and activity. Limited proteolysis has been used previously to distinguish structurally accessible from inaccessible conformations of proteins, revealing function domains [35,36], and to assess protein stability [37]. Despite the utility of the method, no report to date has explored the limited proteolysis of HDAC1.

We sought to investigate limited proteolysis as a means of characterizing HDAC1 structure. As a first step, we performed digestions with increasing concentrations of trypsin using HDAC1 isolated from human Jurkat cells. While HDAC1 was degraded by trypsin, no evidence of distinct fragments was detected under any reaction conditions (Figure 1A). Presumably, HDAC1 degraded to small peptides undetectable by gel electrophoresis (Figure 2). Because HDAC1 degraded without production of discrete fragments, the data suggest that the HDAC1 structure lacks distinct domains of protease-resistance. Rather, the data are consistent with a model where HDAC1 comprises inter-related regions that cooperate to maintain overall structure and function. This model is corroborated by previous work documenting the temperature sensitivity of HDAC1 activity [33].

Previous studies with human SIRT3 and maize Zm-Hda1 demonstrated that post-translational proteolysis gave rise to enzymatically active forms of the proteins [38,39]. Because HDAC1 proteolysis by trypsin did not produce discrete fragments, the results suggest that HDAC1 is not subjected to the same proteolytic processing as SIRT3 and Zm-Hda1. However, it is possible that alternative proteases are required for HDAC1 proteolysis *in vivo*.

Because no discrete protease fragments were detected with HDAC1, we focused the studies on analyzing the concentration-dependent degradation of HDAC1 by trypsin [37]. Since HDAC1 phosphorylation promotes HDAC1 enzymatic activity and protein associations [24], we studied HDAC1 mutants lacking phosphorylation sites. We hypothesized that unphosphorylated HDAC1 mutants would give different concentration-dependent degradation than wild-type HDAC1 if interaction with associated proteins or enzymatic inactivity govern proteolysis susceptibility. We found that HDAC1 phosphorylation site mutants were significantly more sensitive to trypsin digestion compared to wild type HDAC1 (Figure 3), suggesting that HDAC1 trypsin sensitivity correlates with the interaction with associated proteins, enzymatic activity, or both.

To decipher whether sensitivity to trypsin correlates with HDAC1 enzymatic activity and/or protein association, we performed trypsin digestion experiments with a variety of single point mutants- S421A, S423A, E424A, E426A, and H141A. Because each of the single point mutants has a differing enzymatic activity and propensity to interact with associated protein, the combined data was expected to reveal the factors governing trypsin sensitivities. Multiple experiments indicate that the trypsin sensitivity of HDAC1 is not correlated with enzymatic activity. While all single point mutants are less active than wild-type HDAC1, they all demonstrated similar trypsin digestion to wild-type HDAC1. Most significantly, the HDAC1 H141A mutant is inactive due to mutation of a catalytic histidine, yet it displays identical trypsin digestion compared to wild-type HDAC1 (Figure 6). Therefore, the data suggest that the heightened susceptibility of the HDAC1 S421A/S423A mutant to trypsin digestion does not correlate with enzymatic inactivity.

The experiments with the single point mutants are also helpful in illuminating the role of protein association in governing trypsin digestion susceptibilities. Unlike wild type HDAC1, all of the phosphorylation site single point mutants have reduced ability to interact with RbAp48, mSin3, MTA2, and CoREST. Consequently, if associations with RbAp48, mSin3, MTA2, and CoREST dictate trypsin susceptibility, the expectation was that the wild type and single point mutants would display significantly different sensitivities to trypsin. The data indicated that the single point mutants displayed trypsin sensitivities similar to wild type HDAC1-F, with only a modest difference with 1X concentration of trypsin. While differences in trypsin sensitivities at 1X concentration may result from association with RbAp48, mSin3, MTA2, and CoREST, the data indicate that vulnerability to trypsin digestion is independent of RbAp48, mSin3, MTA2, and CoREST at high concentrations of trypsin.

Previous work found that binding with Sin3, RbAp48, MTA2, and CoREST was lost whether HDAC1 was singly or doubly mutated at S421 and S423, conjuring the possibility that phosphorylation at either site is functionally redundant *in vivo *[24]. If singly and doubly phosphorylated HDAC1 proteins are functionally indistinguishable, the expectation was that their trypsin sensitivities would be similar as well. The data indicated that the single and double phosphorylation site mutants demonstrated significant differences in proteolysis sensitivities at all trypsin concentration tested. The fact that the single and double point mutants differ in their protease susceptibilities suggests a model where each phosphorylation event individually contributes to HDAC1 activity. Because HDAC1 associates *in vivo *with multiple proteins, it is attractive to hypothesize that unphosphorylated HDAC (double mutant) is distinguished from singly phosphorylated HDAC1 via differing protein interactions. For example, in addition to interaction with proteins in the Sin3, NuRD, and CoREST complexes [16,18,20,40,41], HDAC1 directly interacts with transcription factors, such as retinoblastoma protein [42], estrogen receptor alpha [43], and Sp1 [44]. While additional studies are necessary to unambiguously identify the phosphorylation-dependent events that correlate with proteolysis susceptibility, the limited proteolysis studies provide evidence that singly phosphorylated HDAC1 is distinguishable from unphosphorylated HDAC1.

The limited proteolysis provides a secondary assay to confirm that mutagenesis does not result in the global instability and unfolding of the protein [24]. For example, the limited proteolysis demonstrated that HDAC1 S421A and HDAC1 S423A maintain similar sensitivities to proteolysis compared to wild type HDAC1 even though they are catalytically inactive and unable to interact with associated proteins. Therefore, limited proteolysis analysis provides a general assay for assessing HDAC1 protein stability.

Previous work showed that phosphorylation at S421 and S423 promotes the full enzymatic activity and protein associations of HDAC1. Explaining these results, two non-mutually exclusive hypotheses were proposed [24,45]. The first hypothesis suggests that phosphorylation at S421 and S423 causes a conformational change, which promotes protein association and augments enzymatic activity. The second hypothesis posits that HDAC1-dependent phosphorylation promotes protein associations, which results in augmented enzymatic activity. While it is formally possible that phosphorylation may result in subtle structural rearrangements undetectable by limited proteolysis, the data are consistent with the possibility that protein associations promote HDAC1 enzymatic activity. A model of associated protein-promoted HDAC1 activity is strengthened by experiments where coexpression of MTA2 with the HDAC1/HDAC2 core complex resulted in augmented deacetylase activity [16]. Therefore, the results with limited proteolysis reinforce the close relationship between protein association and enzymatic activity in regulating HDAC1 function.

## Conclusion

Limited proteolysis experiments were performed with HDAC1 for the first time. Because discrete HDAC1 fragments were not observed upon degradation with trypsin, the data suggest that HDAC1 contains a contiguous structure cooperating to maintain activity. Concentration-dependent proteolysis with HDAC1 phosphorylation site mutants demonstrated that trypsin sensitivities vary with extent of phosphorylation. As a result, these studies provide the first evidence that phosphorylation of S421 or S423 individually contribute to HDAC1 function. Finally, the data are consistent with a model where HDAC1 catalytic activity is promoted by protein associations.

## Methods

### Cell culture

T-Ag Jurkat cells were grown in RPMI containing phenol red indicator, 10% fetal bovine serum and antibiotic (Gibco). To express HDAC1-F or HDAC1 mutants, 40 million cells were electroporated in indicator-free RPMI with 20 μg of the HDAC1 expression construct, as described [24]. After 48 hours of recovery, the cells were washed with PBS buffer (0.02 M sodium phosphate buffer with 0.15 M sodium chloride, pH 7.4) and stored at -80°C.

### Limited trypsin proteolysis

40 million cells were lysed with 1 mL of cold jurkat lysis buffer (JLB: 50 mM Tris (pH 8), 150 mM NaCl, 10% glycerol and 0.5% Triton X-100) in presence of protease inhibitor cocktail (Calbiochem) and 1 mM PMSF. To immunoprecipitate endogenous HDAC1, the lysate was incubated with 1 μL/mL of monoclonal antibody against HDAC1 (Sigma) for 2 hours prior to addition of 40 μL of protein A agarose beads (Sigma). To immunoprecipitate HDAC1-F or HDAC1 mutants, the lysates were incubated with 40 μL of anti-Flag antibody conjugated agarose beads (Sigma) for one hour. The immunoprecipitated HDAC proteins were equally divided into eight reactions, each of which was incubated either in the absence or presence of increasing concentrations of sequencing grade trypsin (Sigma- 0.00063-0.010 mg/mL or 12–190 units/mL) for 10 seconds at room temperature. Fully digested HDAC1 was generated by incubating with 0.05 mg/mL trypsin for 30 minutes at room temperature. The reactions were quenched with SDS loading buffer (0.1 M Tris pH 8.9, 4% SDS, 2 mM EDTA, 0.1% bromophenol blue, and 20% glycerol). After addition of 1 μL of β-mercaptoethanol, the reaction products were separated by SDS-PAGE on a 15% gel. The separated proteins were visualized either by silver staining or western blotting with anti-HDAC1 (Sigma) or anti-Flag (Sigma) antibodies.

### Quantification of trypsin digestion

To create quantitative histograms of the extent of trypsin digestion, the amount of full length HDAC1 protein detected in the western blot was quantified on a Storm 860 Phosphoimager using the program IQMac version 1.2. Briefly, the intensity of each protein band was determined after subtraction of background signal due to nonspecific antibody staining of the membrane. Due to the inhomogeneity of nonspecific antibody staining, a visual inspection of each background correction was necessary to ensure accuracy. To quantify the extent of trypsin digestion, the intensity of full length HDAC1 incubated in the presence of trypsin was divided by the intensity of HDAC1 in the absence of trypsin to yield the percentage of full-length protein. The percentage of full-length protein was plotted versus concentration of trypsin for each tested protein to produce the histogram. The data was fit to a single exponential decay curve [37]. The data represent an average of three independent trials [see Additional Files 3, 4, 5, 6, 7], with standard error indicated with error bars.

### HDAC assays

HDAC1-F was immunoprecipitated and digested with increasing concentrations of trypsin as described. In this case, to maintain HDAC1 in a native form for subsequent HDAC assays, the reactions were quenched by addition of trypsin inhibitor Type I-S (Sigma) in a 1:1 (w/w) ratio with trypsin enzyme. Lower concentrations of trypsin were used in these reactions (0.00007.8-0.0050 mg/mL or 1.4–90 units/mL) because the trypsin inhibitor did not immediately stop the reaction, as was the case with SDS quenching. The HDAC assays were described previously where [^3^H]-acetate incorporated histones (approximately 0.2 μg or 500 dpm) were incubated with the digested protein for 1 hour at 37°C in 50 μL HD buffer (20 mM Tris, pH 8.0, 150 mM NaCl, and 10 % glycerol) [24]. An equal volume of stop solution (0.5 M HCl and 0.2 M acetic acid) was added to quench the reaction and deacetylase activity was determined by scintillation counting of the ethyl-acetate-soluble [^3^H]-acetic acid.

## Authors' contributions

NK performed all proteolysis experiments and data quantification. PK-D contributed the HDAC assay data. MKHP conceived of the study, performed data analysis, and drafted the manuscript. All authors read and approved the final manuscript.

## Supplementary Material

Additional File 1 The predicted fragments produced from digestion of human HDAC1 with trypsin using Peptide Cutter [27]. Table indicating the location of predicted trypsin cleavage sites in HDAC1, along with the expected peptide fragment sequences, lengths, masses, and cleavage probabilities Click here for file

Additional File 2 Percentage of full-length protein remaining after incubation of each wild type or mutant HDAC1 with increasing concentrations of trypsin. Table indicating the percentage full length protein remaining after cleavage, along with standard error, for all trypsin concentrations and all proteins Click here for file

Additional File 3 Limited proteolysis of endogenous and transiently expressed HDAC1. Figure showing all proteolysis experiments with endogenous and transiently expressed HDAC1 used for quantitative analysis, as well as control reactions with anti-Flag-bound solid phase in the presence and absence of trypsin Click here for file

Additional File 4 Limited proteolysis of HDAC1 S421A/S423A and E424A/E426A mutants. Figure showing all proteolysis experiments with HDAC1 S421A/S423A and E424A/E426A mutants used for quantitative analysis Click here for file

Additional File 5 Limited proteolysis of HDAC1 S421A and S423A mutants. Figure showing all proteolysis experiments with HDAC1 S421A and S423A mutants used for quantitative analysis Click here for file

Additional File 6 Limited proteolysis of HDAC1 E424A and E426A mutants. Figure showing all proteolysis experiments with HDAC1 E424A and E426A mutants used for quantitative analysis Click here for file

Additional File 7 Limited proteolysis of the HDAC1 H141A mutant. Figure showing all proteolysis experiments with the HDAC1 H141A mutant used for quantitative analysis Click here for file
