# Supplementary material for: Limited proteolysis of human histone deacetylase 1
Source: BMC Biochem. 2006 Oct 5;7:22. doi: 10.1186/1471-2091-7-22 (PMC1613246; doi:10.1186/1471-2091-7-22)
Supplement: Additional File 1 — The predicted fragments produced from digestion of human HDAC1 with trypsin using Peptide Cutter [27]. Table indicating the location of predicted trypsin cleavage sites in HDAC1, along with the expected peptide fragment sequences, lengths, masses, and cleavage probabilities [file 1471-2091-7-22-S1.pdf]

Additional File 1: The predicted HDAC1 fragments produced from digestion with trypsin

Position	Resulting peptide sequence	Length	Mass	Cleavage Probability
8	MAQTQGTR	8	891.998	90.9 %
9	R	1	174.203	72.9 %
10	K	1	146.189	84.5 %
31	VCYYYDGDVGNYYYGQGHPMK	21	2492.719	22.9 %
34	PHR	3	408.461	100 %
36	IR	2	287.362	100 %
49	MTHNLLLNYGLYR	13	1607.891	84.5 %
50	K	1	146.189	75 %
55	MEIYR	5	710.846	21.3 %
58	PHK	3	380.447	100 %
66	ANAEEMTK	8	892.979	100 %
74	YHSDDYIK	8	1040.097	100 %
77	FLR	3	434.539	100 %
80	SIR	3	374.440	34.9 %
89	PDNMSEYSK	9	1070.139	100 %
93	QMQR	4	561.657	100 %
123	FNVGEDCPVFDGLFEFCQLSTGGSVASAVK	30	3124.487	100 %
126	LNK	3	373.453	100 %
143	QQTDIAVNWAGGLHHAK	17	1846.035	90.5 %
144	K	1	146.189	90.9 %
165	SEASGFCYVNDIVLAILELLK	21	2297.692	100 %
169	YHQR	4	602.651	100 %
192	VLYIDIDIHHGDGVEEAFYTTDR	23	2678.895	100 %
200	VMTVSFHK	8	948.148	100 %
212	YGEYFPGTGDLR	12	1374.472	91.8 %
218	DIGAGK	6	559.620	96.3 %
220	GK	2	203.241	100 %
229	YYAVNYPLR	9	1158.322	91.8 %
242	DGIDDESYEAIFK	13	1501.566	40.6 %
247	PVMSK	5	560.709	100 %
270	VMEMFQPSAVVLQCGSDSLSGDR	23	2456.786	79.4 %
279	LGCFNLTIK	9	1008.244	100 %
283	GHAK	4	411.461	100 %
289	CVEFVK	6	723.885	100 %
306	SFNLPMLMLGGGGYTIR	17	1827.190	100 %
310	NVAR	4	458.518	100 %
342	CWTYETAVALDTEIPNELPYNDYFEYFGPDFK	32	3852.196	100 %
361	LHISPSNMTNQNTNEYLEK	19	2233.437	100 %
363	IK	2	259.349	100 %
365	QR	2	302.333	100 %
371	LFENLR	6	790.917	100 %
403	MLPHAPGVQMQAIPEDAIPEESGDEDEDDPDK	32	3476.699	73.9 %
404	R	1	174.203	83.4 %
412	ISICSSDK	8	851.970	73.9 %
413	R	1	174.203	83.4 %
431	IACEEEFSDSEEEGEGGR	18	1972.966	90.9 %
432	K	1	146.189	100 %
438	NSSNFK	6	695.730	100 %
439	K	1	146.189	87.5 %
441	AK	2	217.268	87.3 %
442	R	1	174.203	84.5 %
444	VK	2	245.322	100 %
449	TEDEK	5	620.614	79.9 %
451	EK	2	275.305	83.2 %
456	DPEEK	5	616.626	87.3 %
457	K	1	146.189	84.6 %
464	EVTEEEK	7	862.889	100 %
466	TK	2	247.294	90.9 %
469	EEK	3	404.420	26.4 %
473	PEAK	4	443.500	100 %
476	GVK	3	302.374	90.8 %
480	EEVK	4	503.553	100 %
482	LA	2	202.253	-

"Position" indicates the amino acid position of the predicted cleavage sites; "Resulting peptide sequence" is the sequence of each fragment predicted; "Length" is the number of amino acids found in each predicted fragment; "Mass" is the weight in Daltons of each predicted fragment; "Cleavage Probability" is the percentage likelihood a cleavage event would occur at that position given the influence of the surrounding amino acids.
