# Supplementary material for: Limited proteolysis of human histone deacetylase 1
Source: BMC Biochem. 2006 Oct 5;7:22. doi: 10.1186/1471-2091-7-22 (PMC1613246; doi:10.1186/1471-2091-7-22)
Supplement: Additional File 2 — Percentage of full-length protein remaining after incubation of each wild type or mutant HDAC1 with increasing concentrations of trypsin. Table indicating the percentage full length protein remaining after cleavage, along with standard error, for all trypsin concentrations and all proteins [file 1471-2091-7-22-S2.pdf]

Additional File 2: Percentage of full length protein remaining after incubation with increasing concentrations of trypsin[a]

HDAC1	0X	1X	2X	4X	8X	16X
wild-type (endogenous)	100%	97 ± 1.2%	83 ± 6.8%	44 ± 14%	26 ± 6.0%	5.6 ± 2.6%
wild-type (expressed)	100%	93 ± 1.3%	71 ± 16%	42 ± 9.3%	24 ± 10%	10 ± 5.6%
S421A/S423A	100%	48 ± 6.0%	26 ± 2.7%	17 ± 7.6%	8.6 ± 3.1%	5.9 ± 2.1%
E424A/E426A	100%	55 ± 6.4%	41 ± 3.3%	14 ± 7.2%	5.3 ± 3.5%	4.6 ± 1.9%
S421A	100%	77± 2.8 %	67 ± 9.1%	36 ± 2.9%	8.6 ± 1.7%	8.7 ± 4.5%
S423A	100%	70 ± 7.0%	50 ± 6.6%	38 ± 3.7%	16 ± 7.9%	5.9 ± 3.9%
E424A	100%	91 ± 2.5%	76 ± 6.5%	56 ± 17%	20 ± 5.3%	5.9 ± 3.0%
E426A	100%	84 ± 1.2%	56 ± 11%	35 ± 6.1%	15 ± 4.9%	11 ± 3.8%
H141A	100%	92 ± 4.1%	59 ± 24%	38 ± 6.5%	22 ± 4.2%	11 ± 7.0%

[a] Percentage of full-length protein remaining after cleavage was calculated for each protein by comparing the amount of full-length protein in the presence of trypsin with the amount observed in the absence of trypsin. The concentration of trypsin is indicated in Figure 1. The percentage standard error was calculated from at least 3 independent trials.
