# Supplementary material for: Limited proteolysis of human histone deacetylase 1
Source: BMC Biochem. 2006 Oct 5;7:22. doi: 10.1186/1471-2091-7-22 (PMC1613246; doi:10.1186/1471-2091-7-22)
Supplement: Additional File 3 — Limited proteolysis of endogenous and transiently expressed HDAC1. Figure showing all proteolysis experiments with endogenous and transiently expressed HDAC1 used for quantitative analysis, as well as control reactions with anti-Flag-bound solid phase in the presence and absence of trypsin [file 1471-2091-7-22-S3.pdf]

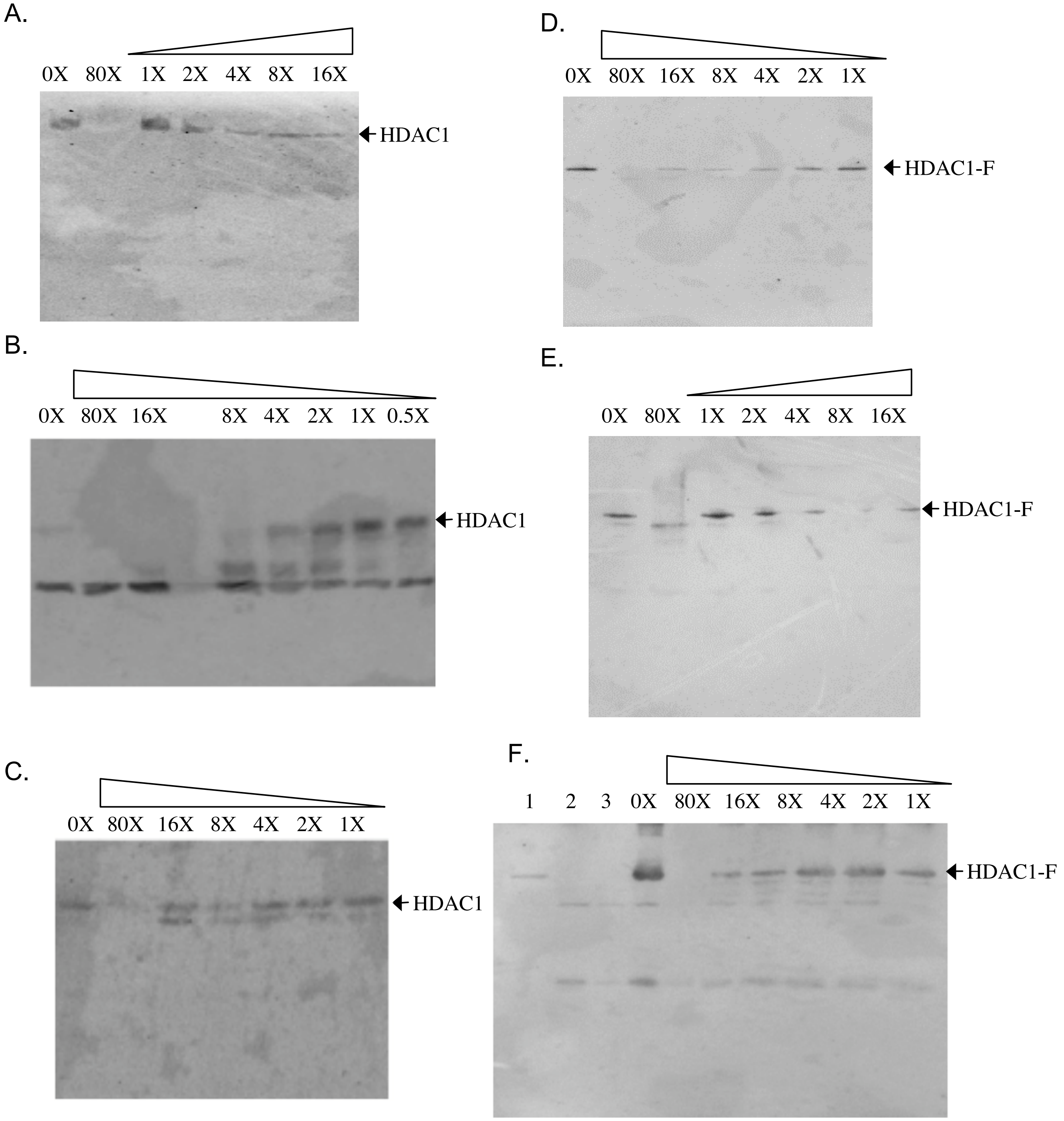

Additional File 3- Limited proteolysis of endogenous and transiently expressed HDAC1
Immunoprecipitated endogenous (A, B and C) and transiently expressed HDAC1-Flag (HDAC1-F) (D, E, and F) were incubated at room temperature with increasing concentrations of trypsin (see Figure 1). After separation by SDS-PAGE, the proteins were visualized with anti-HDAC1 antibody in case of endogenous HDAC1 or anti-Flag antibody in case of transiently expressed HDAC1-F. (F) Additional control lanes are shown- lane 1- TAg Jurkat cell lysates containing expressed HDAC1; lane 2- Flag-Agarose beads alone; lane 3- Flag-Agarose beads incubated with 80X trypsin.
