# Supplementary material for: Limited proteolysis of human histone deacetylase 1
Source: BMC Biochem. 2006 Oct 5;7:22. doi: 10.1186/1471-2091-7-22 (PMC1613246; doi:10.1186/1471-2091-7-22)
Supplement: Additional File 4 — Limited proteolysis of HDAC1 S421A/S423A and E424A/E426A mutants. Figure showing all proteolysis experiments with HDAC1 S421A/S423A and E424A/E426A mutants used for quantitative analysis [file 1471-2091-7-22-S4.pdf]

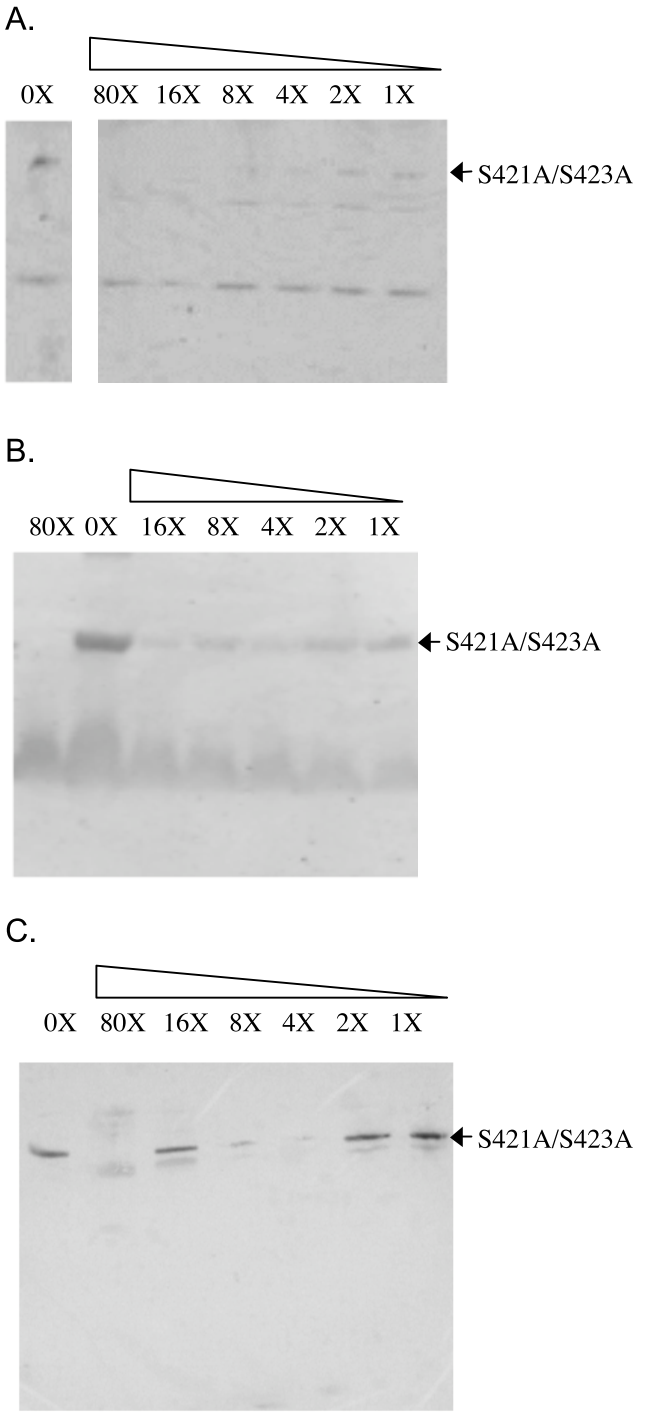

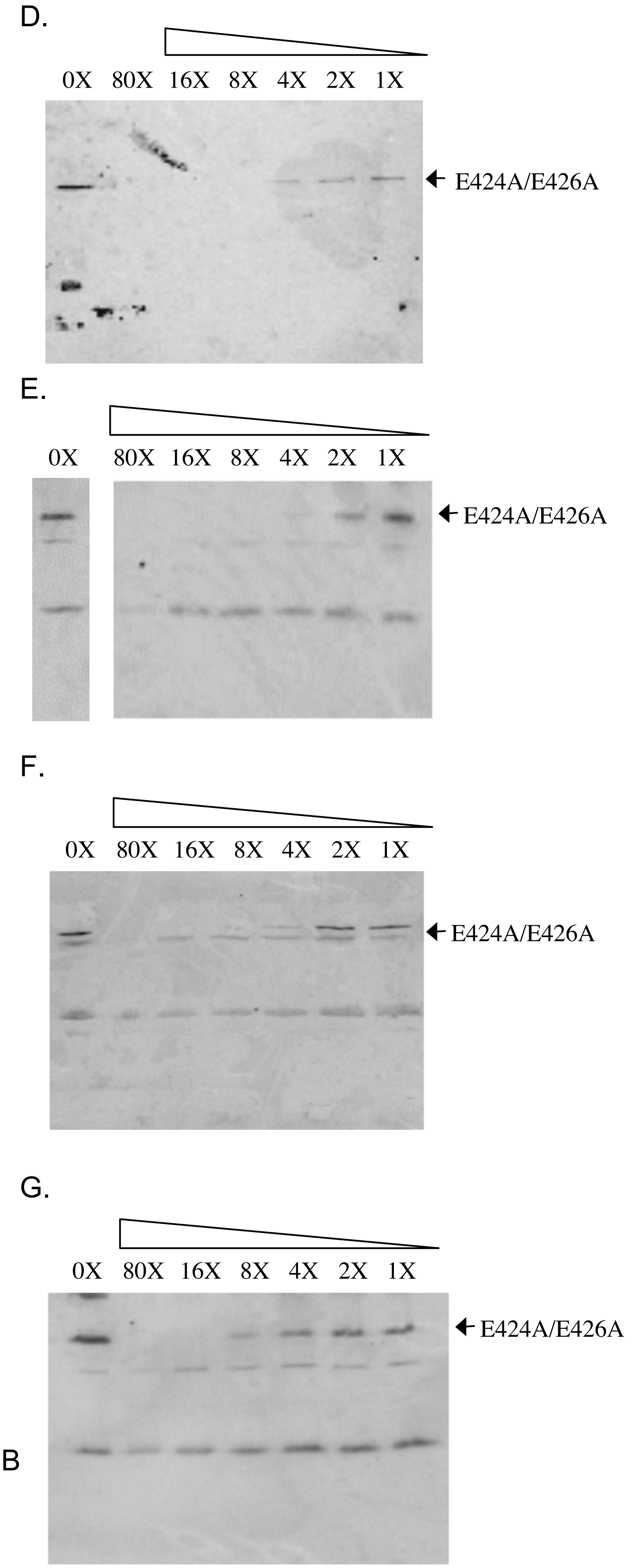

Additional File 4- Limited proteolysis of HDAC1 S421A/S423A and E424A/E426A mutants

Immunoprecipitated HDAC1 S421A/S423A (A, B and C) and E424A/E426A (D, E, F and G) mutatns were incubated with increasing concentrations of trypsin (See Figure 1). After separation by SDS-PAGE, the proteins were visualized with anti-Flag antibody.
