# Supplementary material for: Limited proteolysis of human histone deacetylase 1
Source: BMC Biochem. 2006 Oct 5;7:22. doi: 10.1186/1471-2091-7-22 (PMC1613246; doi:10.1186/1471-2091-7-22)
Supplement: Additional File 5 — Limited proteolysis of HDAC1 S421A and S423A mutants. Figure showing all proteolysis experiments with HDAC1 S421A and S423A mutants used for quantitative analysis [file 1471-2091-7-22-S5.pdf]

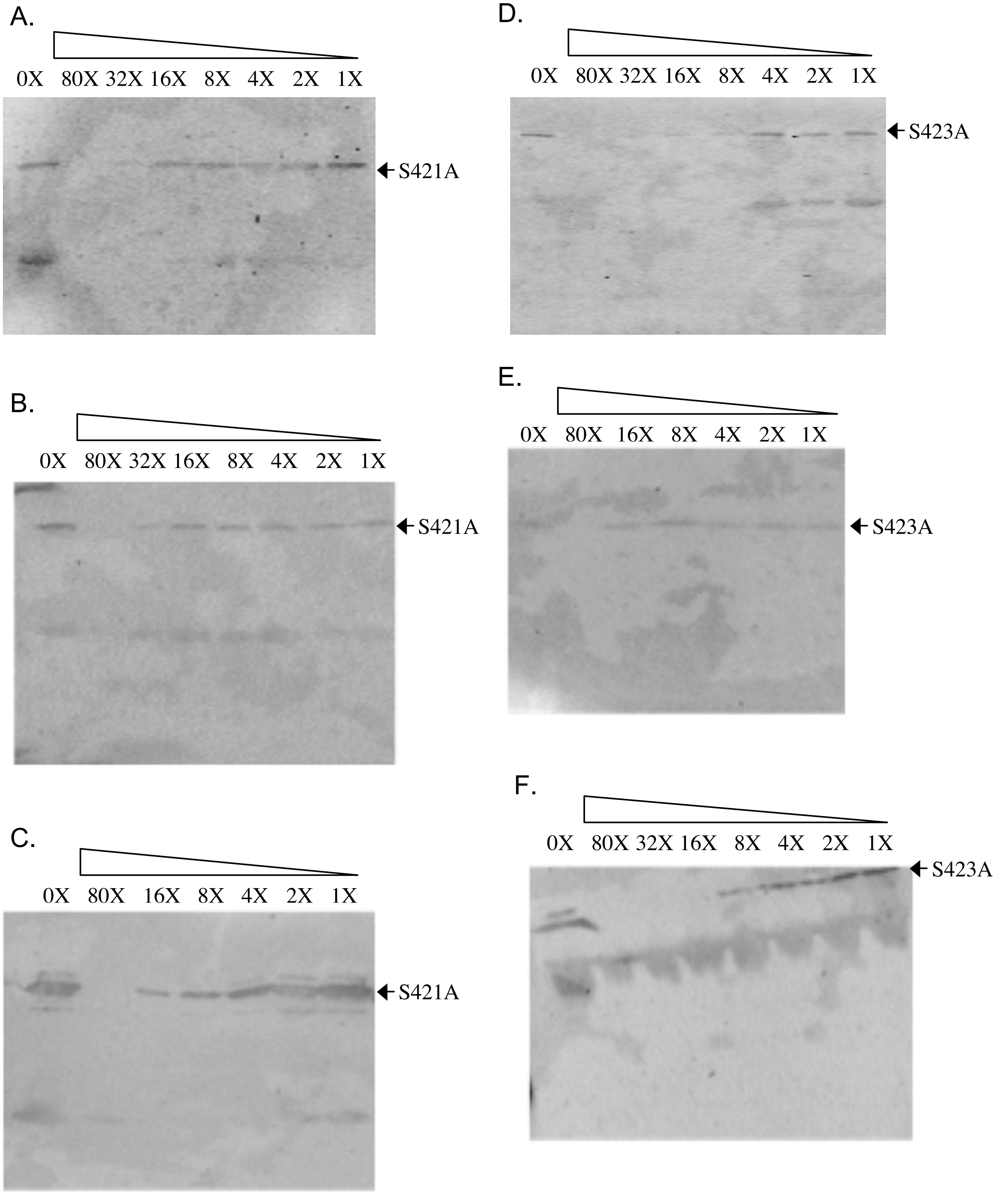

Additional File 5- Limited proteolysis of HDAC1 S421A and S423A mutants
Immunoprecipitated HDAC1 S421A (A, B and C) and S423A (D, E, and F) mutants were incubated with increasing concentrations of trypsin (see Figure 1). After separation by SDS-PAGE, the proteins were visualized with anti-Flag antibody.
