# Supplementary material for: Limited proteolysis of human histone deacetylase 1
Source: BMC Biochem. 2006 Oct 5;7:22. doi: 10.1186/1471-2091-7-22 (PMC1613246; doi:10.1186/1471-2091-7-22)
Supplement: Additional File 7 — Limited proteolysis of the HDAC1 H141A mutant. Figure showing all proteolysis experiments with the HDAC1 H141A mutant used for quantitative analysis [file 1471-2091-7-22-S7.pdf]

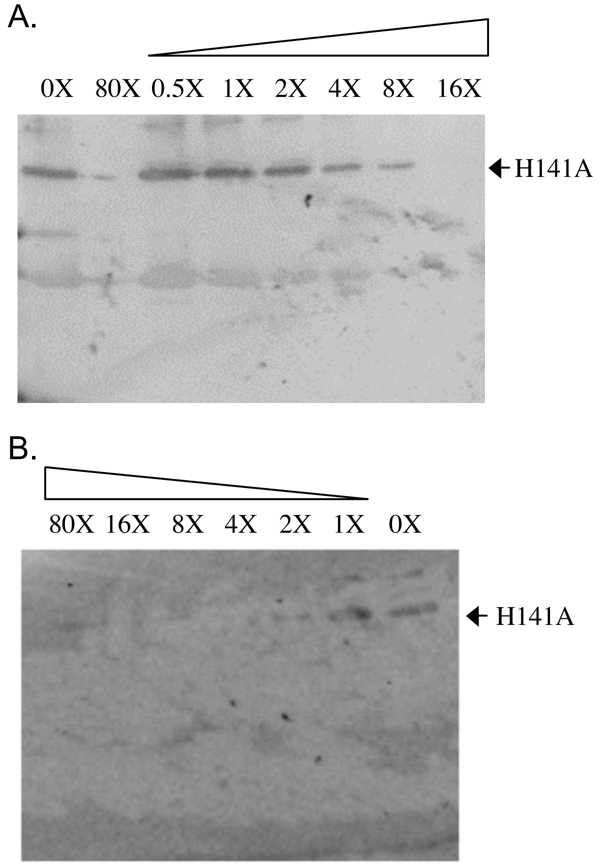

Additional File 7- Limited proteolysis of the HDAC1 H141A mutant

Immunoprecipitated HDAC1 H141A (A and B) mutants were incubated with increasing concentrations of trypsin (see Figure 1). After separation by SDS-PAGE, the proteins were visualized with anti-Flag antibody.
